# Using high-density perimetry to explore new approaches for characterizing visual field defects

**DOI:** 10.1016/j.visres.2023.108259

**Published:** 2023-06-06

**Authors:** I. Marín-Franch, H.J. Wyatt, W.H. Swanson

**Affiliations:** a Computational Optometry, Atarfe, Granada, Spain; b Southwest Eye Institute, Tavistock, UK; c SUNY College of Optometry, New York, NY, United States; d School of Optometry, Indiana University Bloomington, Bloomington, IN, United States

**Keywords:** Visual Function, Micro-perimetry, Angioscotomas, Eye tracking, Information Graphics

## Abstract

High-density threshold perimetry has found that conventional static threshold perimetry misses defects due to undersampling. However, high-density testing can be both slow and limited by normal fixational eye movements. We explored alternatives by studying displays of high-density perimetry results for angioscotomas in healthy eyes—areas of reduced sensitivity in the shadows of blood vessels. The right eyes of four healthy adults were tested with a Digital Light Ophthalmoscope that gathered retinal images while presenting visual stimuli. The images were used to infer stimulus location on each trial. Contrast thresholds for a Goldmann size III stimulus were measured at 247 locations of a 13°×19° rectangular grid, with separation 0.5°, extending from 11° to 17° horizontally and − 3° to +6° vertically, covering a portion of the optic nerve head and several major blood vessels. Maps of perimetric sensitivity identified diffuse regions of reduced sensitivity near the blood vessels, but these showed moderate structure–function agreement that was only modestly improved when effects of eye position were accounted for. An innovative method termed slice display was used to locate regions of reduced sensitivity. Slice display demonstrated that many fewer trials could yield similar structure–function agreement. These results are an indication that test duration might be reduced dramatically by focusing on location of defects rather than maps of sensitivity. Such alternatives to conventional threshold perimetry have the potential to map the shape of defects without the extensive time demands of high-density threshold perimetry. Simulations illustrate how such an algorithm could operate.

## 1. Introduction

Conventional static automated perimetry measures contrast thresholds at discrete locations in the visual field, a spatial undersampling which can cause it to miss perimetric defects (Maddess, 2014). Several labs have shown that denser sampling can reveal defects not found by conventional static perimetry (Numata et al., 2016; Schiefer et al., 2001; Westcott et al., 2002), and can reveal macular defects even in early glaucoma (De Moraes et al., 2017; Hood et al., 2013; Sullivan-Mee et al., 2016). However, denser sampling requires additional testing that either increases test duration or decreases the number of stimulus presentations per location, limiting how much improvement can be obtained by adding locations for thresholding. The current study used threshold perimetry with dense sampling in order to develop alternative approaches for reducing effects of spatial undersampling. These approaches incorporated an aspect of kinetic perimetry that was lost with the move to static automated perimetry.

### 1.1. From kinetic to static perimetry

Perimetry and psychophysics both emerged independently in the middle of the 19th century, with psychophysics concentrating on the fovea and perimetry concentrating on the rest of the visual field. Psychophysics used a wide range of stimuli, often keeping the location fixed in order to allow the large number of stimulus presentations needed for precise quantitative assessment of thresholds. Perimetry measured performance across the entire visual field by moving small stimuli and finding the locations of transitions, called isopters, between the regions where a given stimulus was seen or not seen. Visibility was varied changing the size or contrast of the stimulus, which produced a range of isopters that produced a topographic map of the *hill of vision* (Traquair, 1938). By visual comparison of the isopters found in the two eyes of a patient, a trained clinician could localize the damage, such as before or after the optic chiasm, or in inferior versus superior retina. Evaluations of patterns of damage led to detection of tumors pressing on the optic pathway, vascular damage in the central nervous system, retinal degenerations, and damage to the retinal ganglion cells and their axons from diseases such as glaucoma.

About a century after the emergence of kinetic perimetry, psychophysical techniques were applied to perimetry by measuring contrast thresholds at fixed locations for static presentations of conventional perimetric stimuli (Aulhorn & Harms, 1967; Fankhauser & Schmidt, 1958; Harms, 1952). Static perimetry allowed many fewer locations to be tested, and was originally intended as a supplement to kinetic perimetry for estimating the depth of a scotoma after it had been detected with kinetic testing (Sloan, 1961). However, once static perimetry was automated a larger number of locations could be tested (Heijl, 1976). For manual kinetic perimetry, the perimetrist used information about prior responses to choose the next location to test, the size and contrast of the stimulus, and its speed and direction of motion. The way that these choices were made varied from one perimetrist to another. Furthermore, kinetic perimetry required a skilled perimetrist to identify potential defects for further testing based on rapid changes in sensitivity with location (Lynn, 1969), which led to different visual field locations being tested in different patients. Static automated perimetry offered the possibility of a more uniform approach with the aim to reduce the need for a skilled perimetrist (Fankhauser et al., 1972; Koch et al., 1972) by using fixed grids of locations. For static automated perimetry, a grid of locations is chosen before the examination, and the algorithm selects the next contrast to present based on prior responses.

At first, visual assessment of thresholds obtained from static automated perimetry imitated isopters (Heijl & Krakau, 1975b, 1975a) or used a grayscale at each location to represent thresholds (Chaplin et al., 1973), then used interpolated grayscale images and finally non-interpolated grayscales that represent the threshold as a percentile of the value expected at each location based on age norms and global indices based on those (Flammer, 1986; Heijl et al., 1987). Over time, static automated perimetry replaced manual kinetic perimetry for most clinical uses.

### 1.2. Shortcomings of static automated perimetry

A key issue with the transition from kinetic to static perimetry arose because perimetry relies on visual examination of results, while psychophysics relies on quantitative evaluation of results. Visual examination allows the clinician to identify correspondence with visual examination of the retina or retinal images (originally fundus photos, today from imaging devices). Despite the need for a skilled perimetrist, kinetic perimetry allowed careful evaluation of slow versus rapid changes in sensitivity across the visual field. Static automated perimetry also requires visual examination of patterns of contrast thresholds. However, it is weaker than kinetic perimetry in making this evaluation because of spatial undersampling and high test–retest variability of contrast thresholds in damaged regions estimated from a small number of stimulus presentations.

Quantitative analysis of static automated perimetry found that the substantial between-subject variability in normative databases led to poor structure–function agreement in patients with glaucoma (Ashimatey & Swanson, 2016). Furthermore, the use of normative data led to artifacts such as seemingly better performance for new forms of perimetry that was actually due to differences in how normative data were gathered for the different methods (Sample et al., 2006). Finally, over time it became clear that the high test–retest variability of static threshold estimates, and their low sampling density, made it difficult to reliably visualize the shape of a scotoma. It also became clear that these issues had led to the mistaken idea (Malik et al., 2012; Marín-Franch et al., 2013) that structural damage preceded perimetric damage (Kerrigan-Baumrind et al., 2000; Quigley et al., 1989; Wollstein et al., 2011). Today it is widely accepted that perimetry can sometimes detect glaucoma before imaging (Malik et al., 2012), and that imaging methods are not suitable for mass screening (Gedde et al., 2021).

### 1.3. Modernizing static automated perimetry

The current study was undertaken as a step towards a new approach for static perimetry, not reliant on thresholding. Thus, rather than measuring thresholds at different locations and then comparing these, the goal of the present work was to look for relative changes in sensitivity with location. This was done in traditional kinetic perimetry by a perimetrist who individualized stimulus choice and locations tested with a moving stimulus, but the new approaches would use static presentations with retinal locations and contrasts determined for each subject at runtime by a computer algorithm. In order to establish the basis for such algorithms, in this study thresholds were measured for a dense grid of test locations, and eye position was estimated for each stimulus presentation based on fundus imaging. We evaluated the naturally-occurring deep scotoma at the optic disc, and the adjacent angioscotomas (Remky et al., 1996; Tuten et al., 2012) which are small and shallow depressions in healthy eyes caused by shadows cast by retinal vessels (Evans, 1926; Zulauf, 1990). Angioscotomas have been used more generally to assess effectiveness of different visual stimuli (Schiefer et al., 1999), custom test grids (Benda et al., 1999; Schiefer et al., 2010), and different technology such as micro-perimetry (Remky et al., 1996), fundus-driven perimetry (Schiefer et al., 1999), and adaptive optics (Tuten et al., 2012). Here we use angioscotomas to provide clear structural information about a small and mild scotoma, and the blind spot for a very deep scotoma, so we can test the performance of different approaches for localizing defects.

The individual subject responses to stimulus presentations were analyzed to visualize local depressions in sensitivity, using several different approaches. Simulations were performed for a sample algorithm based on these findings that needed only a few minutes to identify mild perimetric defects. The goal of this work was not to find optimal solutions, but rather to set the foundations for alternatives to high-density perimetry. The methodology proposed here may be used to assess and help develop perimetric methods based on newer technology, particularly strategies to utilize eye-tracking information.

## 2. Methods

### 2.1. Participants

The right eyes of four healthy adults were tested with a DLO-LV (Digital Light Ophthalmoscope-Low Vision, Aeon Imaging, Bloomington IN) (Muller et al., 2015; Muller & Elsner, 2018). This device has a two-way Maxwellian-view optical system with which to present the fixation target and the stimuli on a liquid crystal display (37°×21°, 1920 × 1080 pixels) while simultaneously capturing a continuous stream of retinal images at a frame rate of 25 Hz. This system permitted post-hoc registration of eye position during presentation of perimetric visual stimuli. The ages of these participants, with identification numbers S1, S2, S3, S4, were 32, 57, 30, and 42 years. Three of them were female. They all had best-corrected visual acuity of 20/20 or better. Participant S2 was the only one with astigmatism (−0.75 D at 29°). The spherical refractive errors for the four participants were, respectively, −2.25 D, 0.5 D, −1.75 D, and 0 D, and were accounted for on the DLO by bringing the retina into focus. None of the participants had any systemic disease affecting visual function, history of ocular disease, an intraocular pressure greater than 21 mm Hg for the last clinic visit, or an abnormal appearance of the fundus. The research methods followed the tenets of the Declaration of Helsinki and were approved by the Indiana University Institutional Review Board (IRB). Informed consent was obtained for all 4 participants.

### 2.2. Procedure

Contrast thresholds were obtained for the Goldmann size III stimulus (a circle of 0.43° diameter) using a ZEST algorithm (King-Smith et al., 1994; Turpin et al., 2003) as in Gardiner et al. (Gardiner et al., 2014) at 247 locations of a 13°×19° rectangular grid, separated by 0.5° from 11° to 17° horizontally and from −3° to 6° vertically to cover a portion of the optic nerve head and several major blood vessels. A separation of 0.5° was selected so that separation between targeted retinal patches (ignoring the effects of blur) was at least as large as the stimulus size and because typical eye movements during stimulus presentation are of similar magnitude (Demirel & Vingrys, 1993; Henson et al., 1996). To reduce the likelihood of eye movements during stimulus presentation, stimulus duration was set to 60 msec; this is much briefer than the usual 200 msec presentation but longer than the 99.7th percentile for critical duration (a measure of temporal integration) estimated for healthy subjects for a stimulus of similar size —0.48° diameter (Mulholland et al., 2015a, 2015b). The background luminance was 20 cd/m^2^, the maximum luminance increment was 80 cd/m^2^, and the maximum contrast was 400%. The ZEST algorithm used a total of 74 different Weber contrasts from − 0.9 to + 0.6 log unit, with a median difference of 0.02 log unit between contrasts. The algorithm terminated at a given location after six presentations. The interstimulus interval averaged 1200 msec, with a variable foreperiod.

Contrast thresholds were measured at the 247 retinal locations in 8 test blocks, each block testing between 27 and 40 locations. Each block used a grid of locations with 2° spacing horizontally and vertically, such that the 8 blocks combined yielded a grid with 0.5° spacing. For each of the subjects recruited for this experiment, all 8 test blocks were taken on the same day. The ZEST algorithm used 6 stimulus presentations per location. Test duration averaged 4 min and 58 s per block, with a standard deviation of 51 s.

False positives and false negatives were assessed as in previous studies (Hot et al., 2008; Keltgen & Swanson, 2012), but unlike in previous studies, fixation losses were not assessed because eye position was inferred from the retinal images recorded by the DLO system. Three images were used to register eye position at each stimulus presentation. An image was acquired every 40 msec. Therefore, two images were acquired during the 60 msec of stimulus presentation and one after it. Eye displacement in camera pixels was determined for each stimulus presentation post-hoc from the three images. The relative displacement was estimated for each image as relative displacement of the optic disc from a reference image, and the medians in *x* and *y* were used as the estimate for the trial. Eye displacement in degrees of visual angle was obtained by multiplying the estimated displacements in camera pixels by the angle subtended by one pixel, 1.64 arc min (0.027°).

For each image, the location of the optic disc was identified by image-processing and was used to quantify eye displacement. Trials were removed from analysis where one or more of the 3 retinal images for a trial was obscured by a blink, or when a large eye movement meant that the optic disc was not within the image. Microsaccades during fixation of a point in a blank field occur at a frequency of approximately 0.8 per sec (Otero-Millan et al., 2008), so the likelihood of finding one during any 120 msec set of 3 frames is approximately 10%. Finally, trials were removed when the displacement varied by more than 0.5° among the 3 frames, or when any frame was shifted by more than 1° with respect to the reference image. In total, 15.8 %, 8.8%, 7.5%, and 6.4% of trials were removed for S1, S2, S3, and S4, respectively.

### 2.3. Data display

We displayed the high-density perimetry data in three different ways, the first two using thresholds. For all displays, results were superimposed on the enface retinal images to assess structure–function agreement in terms of whether angioscotomas revealed by high-density perimetry corresponded to locations of the major vessels on the retinal images.

#### 2.3.1. Conventional threshold display

The first approach was a grid of conventional thresholds at the nominal locations, without consideration of eye movements. For this display, the contrast threshold at each location was estimated by the ZEST algorithm, and the reciprocal of contrast threshold was plotted as contrast sensitivity in log units. In the second and third displays, we incorporated the retinal position where the stimulus was projected (as opposed to the nominal stimulus location), as determined by the eye-position data.

#### 2.3.2. Maximum-likelihood threshold display

The second approach was a map generated by local maximum-likelihood estimation of contrast thresholds at closely-spaced nominal estimation locations, separated by 0.05° from 11° to 17° horizontally and from −3° to 6° vertically. The threshold for an estimation location used all stimulus presentations within 0.5° of the location. At each estimation location, a weighted maximum-likelihood fit of the data was made using Quick’s version (Quick, 1974) of the Weibull function defining the probability of detecting the stimulus (frequency-of-seeing curve). We modified a procedure described in Swanson and Birch (Swanson & Birch, 1992) for local contrast threshold and slope estimation after fixing false-positive and false-negative rates at 0.02. The modification was that a weight was assigned to each sample point within the 0.5°-radius in a similar fashion as in kernel-density estimation (Silverman, 1986), using a Gaussian kernel with a standard deviation of 0.5° to set the relative weight of each sample point as a function of its Euclidean distance from the estimation point. This way, the contributions of sample points further away were smaller than those closer to the estimation point. The local contrast threshold and slope estimates for a location were those that maximized the value of the weighted maximum-likelihood function at that location. The display was a map of contrast sensitivity, in log units.

#### 2.3.3. Slice display

**Fig. 1.**
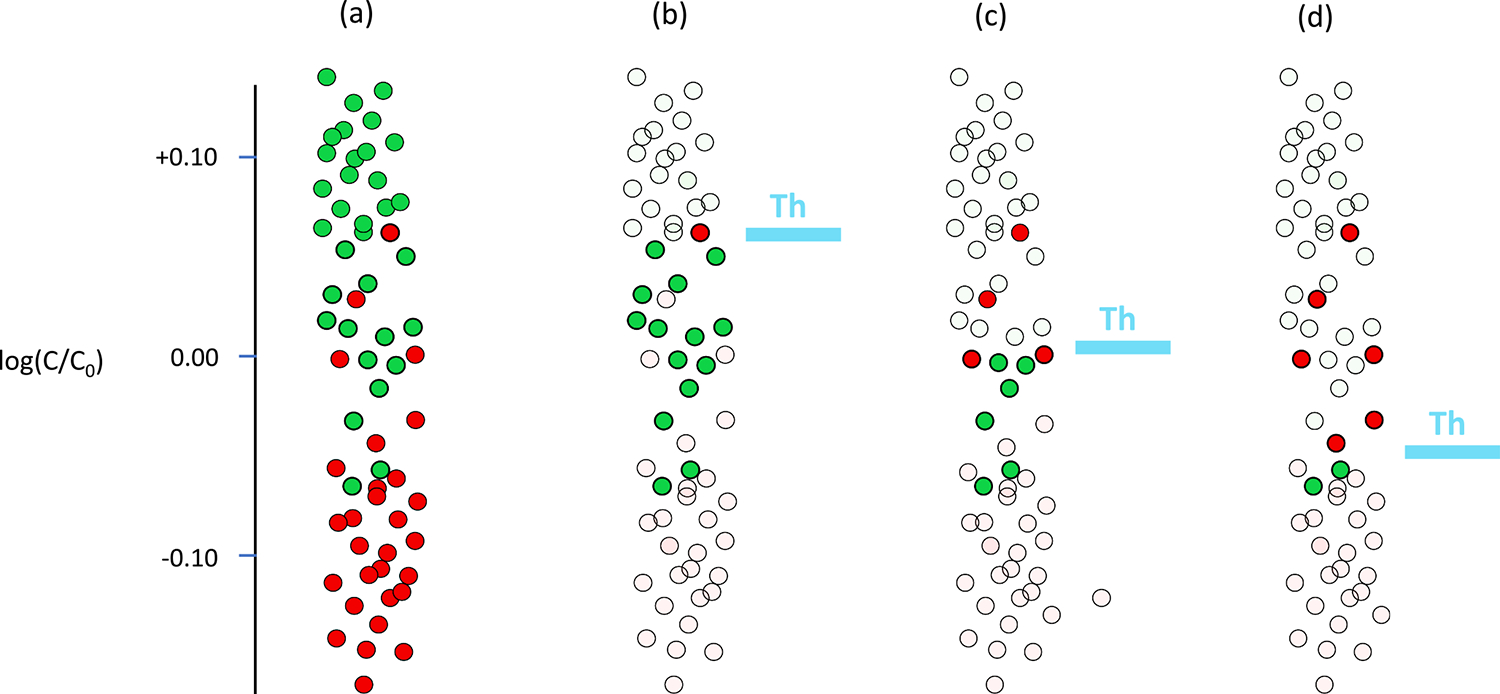
Slice display for hypothetical responses to stimuli at a range of contrasts. The vertical axis shows log contrast of the visual stimuli presented. The green solid circles indicate stimuli for which the participant responded whereas the red solid circles indicate stimuli for which the participant did not respond. The left column (a) shows results for all trials in that patch of retina. The other three columns (b, c, and d) show the partition and behavior of the slice display for three different display thresholds represented by the blue horizontal lines; the pale symbols indicate response trials ignored by the slice display for each position of the display threshold. The slice display ignored trials where the contrast was lower than display threshold and the participant did not respond, as well as trials where the contrast was higher than display threshold and the participant did respond.

The third approach consisted of a *slice display* of a participant’s responses to a set of stimulus presentations, which is described in detail in the Appendix in the supplementary materials. Fig. 1 illustrates the principle with a hypothetical group of responses to a range of stimulus contrasts across a retinal region. Rather than combining responses from different stimulus presentations to estimate threshold, the operator sets an adjustable *display threshold* and the only presentations for which results are shown are where either 1) the stimulus contrast was less than the display threshold yet the participant responded (green circles), or 2) the stimulus contrast was greater than or equal to the display threshold yet the participant did not respond (red circles). Panel (a) shows contrast of each presentation (C) relative to the display threshold (C_0_) for stimulus presentations at different contrasts (vertical displacement), with color indicating the subject’s response to the presentation. Panels (b) to (d) show the points selected for slice display at different threshold values. As the display threshold changes from (b) 0.05 log units to (c) 0.00 log units and to (d) − 0.05 log units, there is a decrease in the number of trials with contrasts less than display threshold for which the participant responded (solid green circles) and an increase in the number of trials for which contrast was greater than the display threshold and the subject did not respond (solid red circles). Thus, if in a region of the retina the local contrast threshold was lower than the display threshold as in panel (b), then most points displayed would be *seen* trials. If the local contrast threshold was greater than the display threshold as in panel (d), then most points displayed would be *not-seen* trials.

The standard definition of threshold is a contrast that is seen 50% of the time. If all locations in a region of retina had the same threshold, then the red and green locations would be expected to be randomly distributed across the retinal region regardless of the choice of display threshold. However, if some locations had elevated thresholds, then with appropriate choice of display threshold the red locations would be of higher density at those locations. With the slice display, it is possible to carry out a visual search for the display threshold that best discriminates retinal patches with depressed sensitivities.

#### 2.3.4. Simulations

These simulations were developed to simulate performance of an algorithm that searches for local declines in perimetric sensitivity. The inputs to the simulations consisted of the results obtained for these four subjects using the maximum likelihood threshold display (section 2.3.2) and the slice display (section 2.3.3). The purpose was to assess how repeatable the structure–function agreement could be for an algorithm that uses a relatively small number of trials to search for mild scotomas. The algorithm assumes that the sensitivity in this region has already been determined. For each test location, the simulations used a frequency-of-seeing curve and a uniform random number generator to determine whether the simulated observer responded to the stimulus presentation. The frequency-of-seeing curve at each location was determined from the subject’s data using the maximum-likelihood estimation method described in section 2.3.2.

Each simulation used a fixed contrast to search for angioscotomas. The contrast used for each simulated observer was set at the contrast that showed the best discrimination of the angioscotoma upon visual inspection of the slice display of data for that subject (see section 2.3.3). Because we were looking for very small scotomas, the algorithm used locations on circles of increasing radius (0.5° steps) from the center of the blind spot. Locations in each circle were separated by approximately 1°. The algorithm started at locations that were not expected to be seen and moved in waves: the closest circle to the center of the blind spot was used for wave 1, the second closest circle for wave 2, and so on. This was possible because the simulations were for testing the blind spot and nearby angioscotomas, so this algorithm is specific to this type of scotoma and is not intended to be generalized.

Once all of the locations in wave 1 had been tested, the algorithm selected for testing only locations in the circle of wave 2 that were neighbors to those in wave 1 for which at least one stimulus presentation had been missed. Neighboring points were defined using Voronoi tessellation. Then, before moving to wave 3, testing was performed at any non-tested points in wave 2 that were neighbors to a tested location with at least one miss. Locations for wave 3 and subsequent waves were chosen using the same procedure as for those of wave 2. The algorithm ended automatically once the edge of all scotomas was found or the algorithm went out of range (i.e., it reached the boundaries of the 13 × 19 test area). The edge of the scotoma was defined as reached if the participant responded both times to the stimulus presented at this location and thereafter no new locations were opened for testing.

The software for data analysis and the example algorithm was written in R (R Core Team, 2022). The data and an app based on the shiny R package (Chang et al., 2020) that allows the analyses to be replicated with different settings can be found as part of the supplementary materials of this manuscript.

## 3. Results

**Fig. 2.**
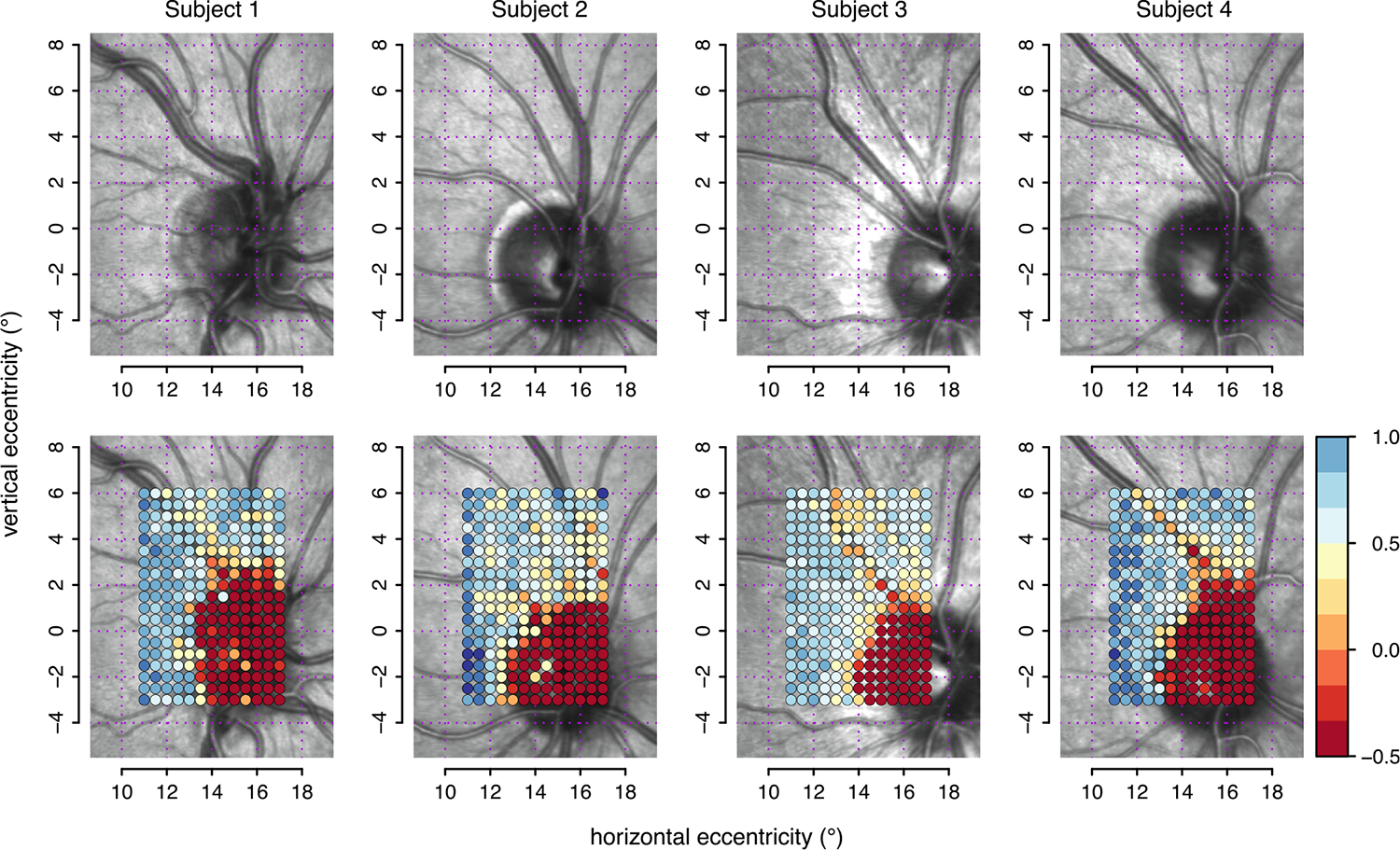
Angioscotomas revealed by conventional threshold display without accounting for eye movements. Upper panels show the enface images for each of the four observers. Lower panels show the corresponding contrast sensitivities at each location as returned by the ZEST algorithm for each nominal location, superimposed on the enface image. The color coding represents the relative contrast sensitivity estimated at each nominal location (circles) from −0.5 to 1.0 log units, with red indicating less-sensitive locations and blue indicating more-sensitive locations.

Fig. 2 shows the region of interest in the fundus images (top row) for the four control subjects and the results from high-density perimetry, using a conventional threshold display at each location without accounting for eye movements.

**Fig. 3.**
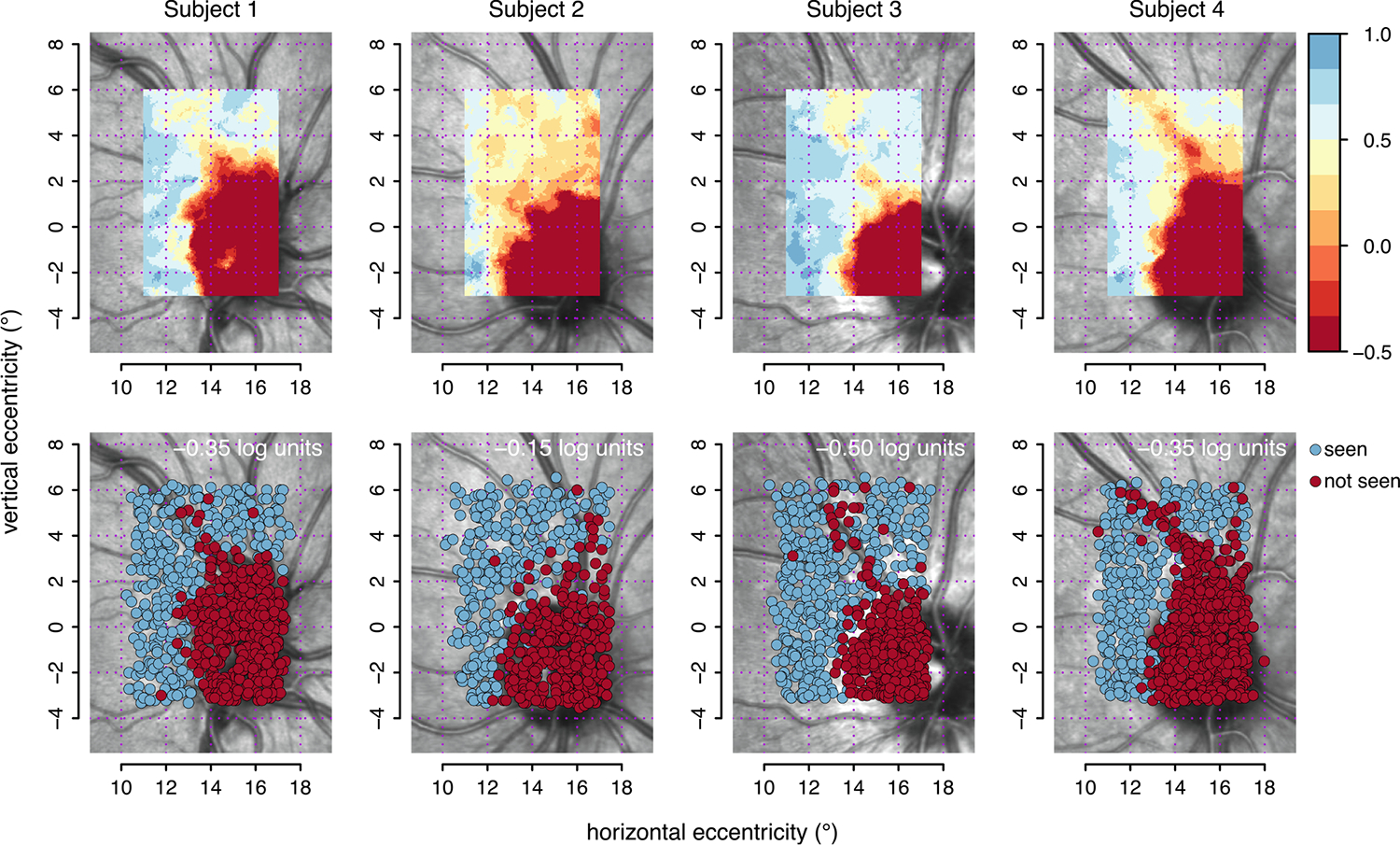
Angioscotomas revealed by local maximum-likelihood sensitivity estimates and the slice display, accounting for eye movements. Upper panels show displays of the local threshold estimates. The color scheme is the same as for Fig. 2. The bottom row shows results for the slice display. Seen trials are shown in blue whereas not seen trials are shown in red.

Fig. 3 shows the results of the second and third analyses. The top row shows results from the local weighted maximum-likelihood estimates of contrast thresholds, taking eye movements into account (section 2.3.2). The bottom row shows the results from the slice display (section 2.3.3). The display threshold for each subject was selected after performing a sweep over a range of contrast values from 0.5 to −0.5 log units in steps of −0.01 log units. For this figure, the display threshold was selected for each subject such that the angioscotomas were revealed most clearly for this individual as judged by the authors. Four videos showing the slice display for each participant and for the range of display thresholds are available as supplementary material to this manuscript.

**Fig. 4.**
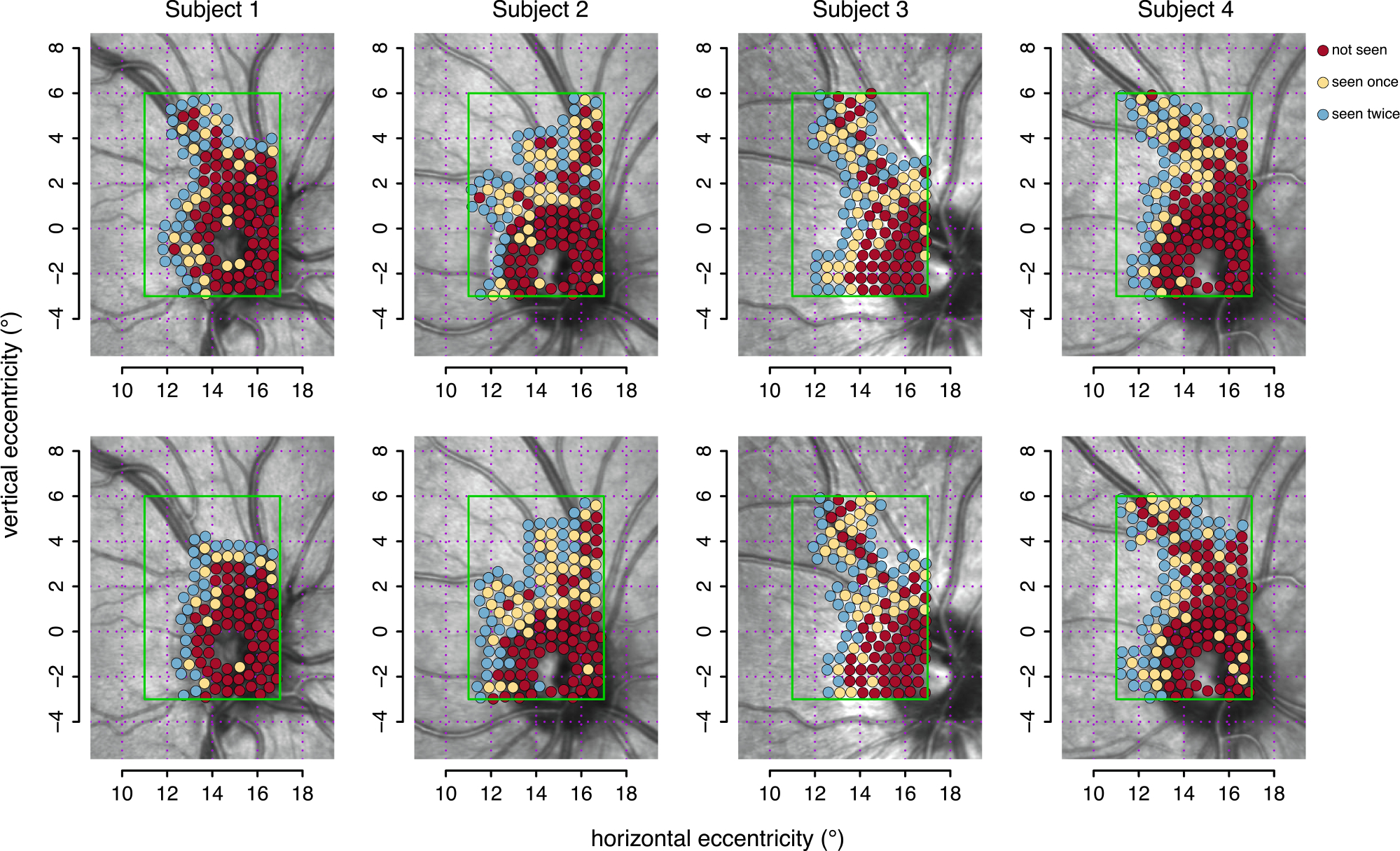
Two sets of simulations of an adaptive algorithm based on the results in Fig. 3. The algorithm used a single stimulus contrast for each subject, the display threshold visually selected in the slice analysis as shown at the top right of the graphs in the lower panel of Fig. 3. Simulated responses for each subject were obtained from the local maximum-likelihood sensitivity estimates shown in the upper panel of Fig. 3. Each location was tested twice. Points shown in blue are locations for which both presentations were seen. Points in yellow represent locations for which one of the two presentations was missed. Points in red represent locations where both presentations were missed.

Fig. 4 shows results of two sets of simulations for each subject. The contrasts, obtained from the slice analysis in Fig. 3, were − 0.35, − 0.15, − 0.50, and − 0.35 log units for Subjects 1 to 4. Two simulations were performed for each subject. The number of locations tested in each simulation were 139 and 110 for Subject 1, 148 and 153 for Subject 2, 138 and 138 for Subject 3, and 156 and 162 for Subject 4. Since there were two stimulus presentations for each subject and location, and assuming an average of 1 sec between each trial, it would take a subject between 4 and 5 min to perform the entire test.

## 4. Discussion

Static automated perimetry has replaced manual kinetic perimetry as a clinical tool for assessing scotomas, but spatial undersampling and variability in threshold measurements limit the ability to visualize the shapes of scotomas. This study explored alternative approaches for assessing scotomas with static perimetry using a principle from kinetic perimetry, that a rapid decrease in contrast sensitivity with stimulus location is an indication of the border of a scotoma. We analyzed results for a large number of stimulus presentations and contrasts in a small retinal region to assess borders of shallow scotomas of limited extent, and found that by applying this principle, it is likely that a much smaller number of presentations could be used to locate these scotomas. We infer that appropriately chosen stimulus contrasts could be used with locations chosen in an adaptive manner to identify scotoma borders, without thresholding. Simulations were performed to demonstrate how such an algorithm might behave over a small retinal region (Fig. 4). Such an approach would only be feasible clinically when used for relatively small retinal regions, as a means to map out specific vulnerable regions, regions with suspected damage, or to corroborate functional damage associated with structural damage seen in enface images. Clinical perimetry typically uses locations across a region with radius of 24°, which would require more than 7000 locations with a separation of 0.5°, or about two hours of testing with a single presentation per location. Furthermore, the large between-subject variability in the shape of the visual field (Swanson et al., 2017) would make analysis of high-density perimetry even more challenging than for conventional perimetry.

We used post-hoc analysis of eye position to assess results of high-density perimetry for the very small and shallow scotomas near the blind spot that are caused by retinal vessels. A dense grid was used with 247 locations and 0.5° spacing, requiring eight testing sessions, many more than could be practical clinically. Even with this very large number of locations with close spacing, the conventional approach for results of high-density perimetry showed relatively diffuse scotomas, due to the effects of eye movements. This represents modest structure–function agreement, where the fundus image indicates the general region where decreased sensitivity can be found but for only one of four subjects did a region of reduced sensitivity align well with a blood vessel. This is illustrated in the bottom panels of Fig. 2, and is consistent with previous observations (Schiefer et al., 1999), including a reported blurring of the angioscotoma edges possibly due to unstable eye fixation (Benda et al., 1999). When information about eye position was used for threshold analysis, there was at most a modest improvement (upper panel of Fig. 3). The angioscotomas shown by the maximum-likelihood threshold estimates do not look very deep and are probably spatially greater than they really are. This is a consequence of the spatial smoothing (of 0.5° of radius) required to obtain the local estimates.

**Fig. 5.**
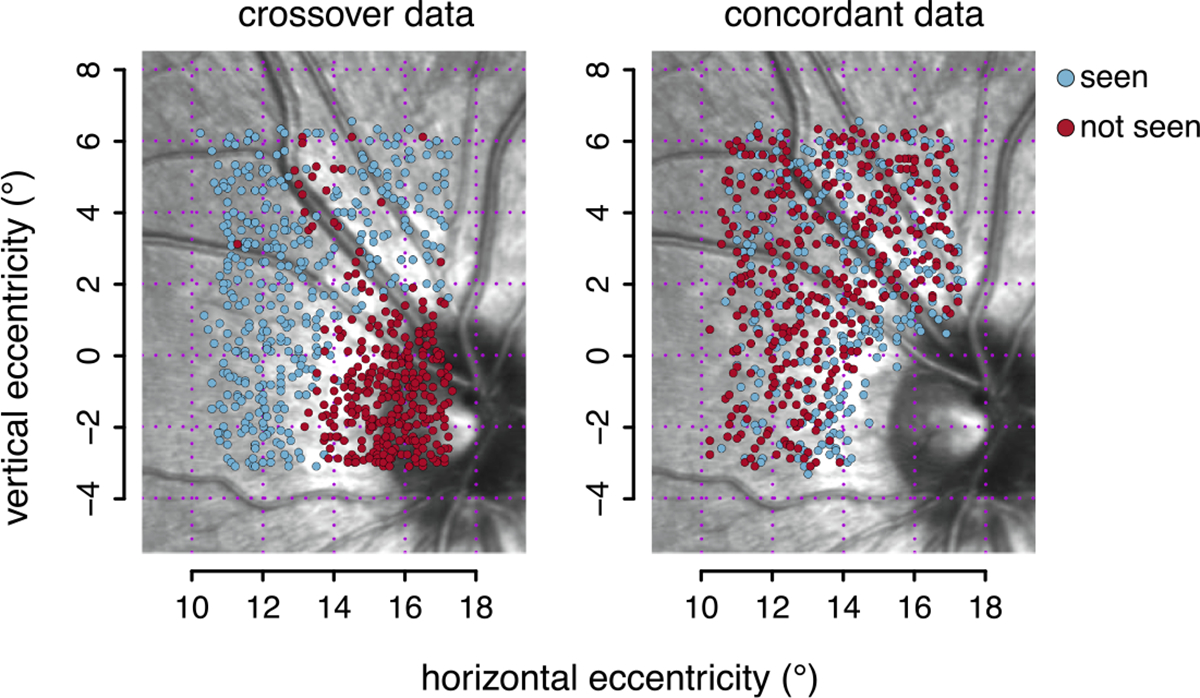
Slice analysis of concordant vs. crossover data, for Subject 3, using a cutoff contrast of −0.50 log unit. The left panel shows the crossover data, that is trials with a contrast greater than −0.50 log units where the stimulus was not seen (red) and trials with a smaller contrast where the stimulus was seen (white). The right panel shows the complementary and mutually exclusive concordant data; that is, trials with a contrast greater than −0.50 log units where the stimulus was seen (white) and trials with a smaller contrast where the stimulus was not seen (red).

The information about eye position allowed us to develop an alternative approach that does not required pooling information of neighboring stimuli location. We termed this new approach a *slice display*, because the use of an adjustable display threshold and the use of only crossover data, result in a type of *slice* of the data, as is apparent in Figs. 1b to 1d. We found that the slice display worked at least as well as threshold estimation (lower panel of Fig. 3). For this slice visualization, we presented the subset of *crossover* presentations for which the subject responded but were smaller than the display threshold and those for which the subject did not respond but were greater than or equal to the display threshold (see section 2.3.3). To illustrate how apparent a defect can be to someone looking at the data. Fig. 5 shows the *crossover* datapoints on the left panel, for the particular value of display-threshold selected for this subject. The right panel of Fig. 5 shows the reduction in datapoints that is produced by suppressing the display of the rest of (*concordant*) datapoints; that is, trials for which the subject did not respond and were smaller than the display threshold and those for which the subject responded and were greater than or equal to the display threshold. Generally, attention is focused on crossover data (See the Appendix in Supplemental materials for details.).

New perimetric approaches could be devised which utilize insights from the slice display, because threshold shows minimal variation with location over small regions in visual space unless there is a scotoma. This means that a relatively narrow range of contrasts would be sufficient to determine whether there is a scotoma in the region. The simulated algorithm (Fig. 4) used a single contrast, but in practice an algorithm that uses more than one contrast would need to assess what contrast range to use in a given region. The low end of the contrast range could then be used in the slice display to plot trials responded to, and the high end would be used to plot trials not responded to. To illustrate the principle, consider a region with a scotoma in which contrast threshold is 0.3 log unit greater than average threshold. Frequency-of-seeing curves in healthy eyes are steep enough (Wall et al., 2002) that a contrast equal to the normal threshold for the region would rarely be responded to at locations within the scotoma, but would be seen for 50% of trials at locations with normal sensitivity. Trials for which this contrast elicited a response would identify regions without a scotoma. By comparison, a stimulus contrast that was 0.3 log unit greater than the average threshold would rarely fail to elicit a response in normal regions, but would fail for 50% of trials at locations in the scotoma. Trials for which this contrast failed to elicit a response would identify regions within the scotoma. This example illustrates how a relatively limited range of contrasts could be used to map a mild scotoma, but does not indicate how an estimate of local threshold would be obtained in the first place. Normative values obtained for healthy eyes could provide a starting point, but would need to be adjusted because of psychophysical factors (criterion, learning, and fatigue) and optical factors (e.g., lens opacity due to cataract). Several methods have been developed to make such adjustments (Åsman et al., 2004; Marín-Franch & Swanson, 2014).

A strength of these findings is that a large amount of data was gathered from a small retinal region in each subject. This enabled comparison of different possible approaches for displaying the results. Another strength is the use of fundus imaging to assess eye movements during perimetric testing and a post-hoc analysis rather than an attempt to compensate for eye movements in real time. Real-time correction for eye position could be used for new algorithms as in Fig. 4, but this is inherently limited for at least two reasons: eye movements can occur during image acquisition, and it takes computing time to register eye position and correct for it, which means that retinal position may be slightly different from the intended position. Post-hoc analysis of eye position can determine whether the eye has moved during stimulus presentation and can identify what retinal location(s) the stimulus fell on.

A limitation is that the study was conducted only on four eyes, and the angioscotomas were small and shallow enough that there were not dramatic differences in structure–function agreement for the different display methods. Another limitation is that the post-hoc estimation of eye position was automated and its accuracy was not quantified. Visual examination of its performance was conducted by observing videos with inferred location overlaid on each frame. While it was observed that the locations usually tracked changes in eye position well, it was noted that in extreme cases the inferred locations could be imprecise by as much as a half a degree. Given that half a degree is the approximate width of the vessels causing the scotomas, this may be one reason why performance was only moderate.

If an algorithm is looking for defects that are much deeper and wider than angioscotomas, the basic approach of searching for local regions of lower sensitivity does not need to rely on measurement of eye position. For this purpose, it could potentially be used in systems that do not provide eye position information (Ashimatey et al., 2021). However, for detecting milder defects that occur at earlier stages of disease, evaluation of eye position may be quite important.

## Supplementary Material

MMC4

MMC5

MMC3

MMC2

MMC1

## Data Availability

The data analyzed in this manuscript and an R app that allows interested readers to replicate our analyses can be found in the supplemental materials https://doi.org/10.1016/j.visres.2023.108259.
