# Supplementary material for: Using high-density perimetry to explore new approaches for characterizing visual field defects
Source: Vision Res. Author manuscript; Available in PMC 2023 Sep 27. (PMC10526895; doi:10.1016/j.visres.2023.108259)
Supplement: MMC5 [file NIHMS1906522-supplement-MMC5.pdf]

APPENDIX -- Using *Crossover* Data for an Interactive Slice Display of Visual Field Data

The purpose of this Appendix is to demonstrate the way in which the slice display operates, and the advantage of focusing attention on "crossover" data.

Consider a 2D region of interest in the visual field. The region can be approximated as a grid of small square areas, where each area is approximately uniform in sensitivity. The sensitivity in each area can be modeled by a sigmoid function describing the probability of seeing a stimulus as a function of stimulus contrast. The upper panel of Figure A1 shows an example of a sigmoid function centered at zero. (The axis can be interpreted as log stimulus contrast relative to some particular contrast.) The lower panel of Figure A1 shows the slope (first derivative) at each point of the sigmoid function in the upper panel; this is maximal at the center. The faster the increase in probability of seeing in the upper panel, the narrower and taller the bell-shaped curve in the lower panel. For areas with greater or lesser sensitivity, the sigmoid is shifted left or right, respectively. For simplicity, the same sigmoid will be used for the entire area of interest but shifted to describe the sensitivity in different local areas.

The height of the sigmoid function at a given contrast is the probability that a stimulus with that contrast will be seen.

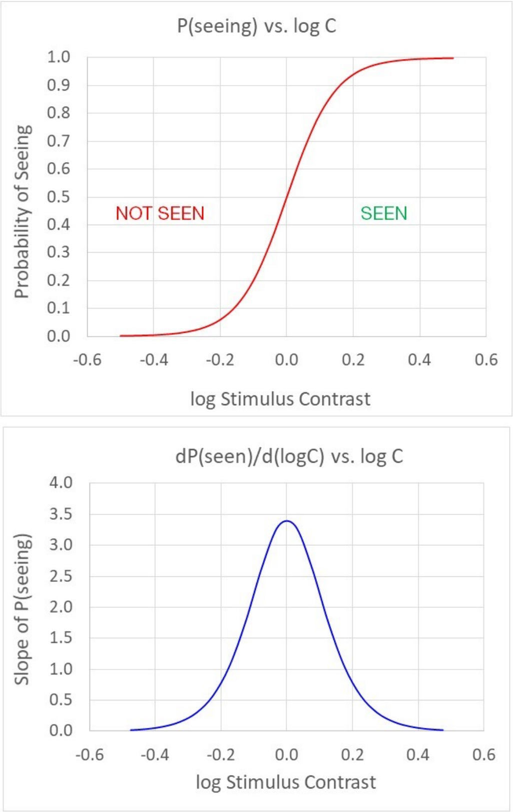

Figure A1

The Slice Display

In a slice display, the data are partitioned with an adjustable parameter called "display threshold" (T.d). The process of partitioning the data is shown in Figure A2. The top row shows the same sigmoid function with the addition of the display threshold, indicated by the blue triangle and dashed line. On the left side of Figure A2, T.d = 0.0; on the right side, T.d = 0.1. In addition to the partitioning of the plot into areas above and below the sigmoid, the display threshold partitions it into contrasts below and above T.d. The region is therefore partitioned into four (2x2) sub-regions, which will be referred to as follows:

Concordant Not-seen (above the sigmoid and < T.d)	*Crossover Not-Seen* (above the sigmoid and > T.d)
Crossover-Seen (below the sigmoid and < T.d)	*Concordant Seen* (below the sigmoid and > T.d)

The arrangement of the terms, above, reflects the position of the four portions of the probability-contrast plots at the top of Figure A2. When the display threshold is near the center of the sigmoid describing sensitivity in an area, the crossover sub-regions (colored orange) are generally much smaller than the concordant sub-regions (colored green) for reasonable sigmoid slopes. Moreover, the crossover sub-regions become progressively smaller than the concordant ones as the sigmoid becomes steeper.

There are two things to keep in mind when looking at the diagrams of Figures A1 and A2: first, each small grid area in the region of interest is associated with a probability diagram of the same type as in Figure A2. Secondly, any individual trial is neither intrinsically crossover nor concordant; for example, a *seen* trial will be concordant when T.d is set below the stimulus value used for that trial, but the same trial will be crossover when T.d is set above the stimulus value.

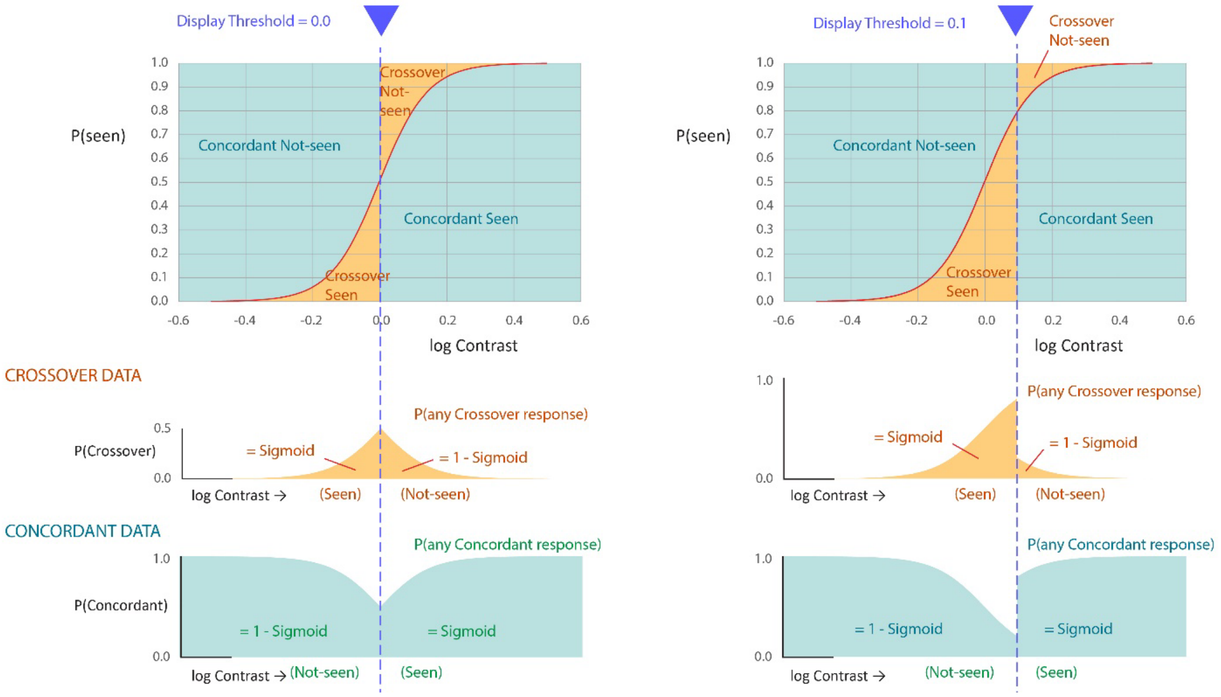

Figure A2

In the lower left portion of Figure A2, the four subregions of the probability diagram (shown at top left) have been separated and the two crossover areas (orange) have been placed on a single horizontal axis so the areas of each may be compared. Similarly, the two concordant (green) areas have also been placed on a single horizontal axis. As must be the case, if the crossover and concordant areas are added together at each contrast, the resulting sum is a rectangle of height 1.

On the right side of Figure A2, the display threshold has been set to 0.1 log units above the center of the same sigmoid. The result is a dramatic shift in the balance of the two crossover subregions, the *not-seen* area decreasing and the *seen* area increasing. The shifts in relative area reflect the shifts in probability, especially for stimulus contrasts near the sigmoid center. *This property is the key to the capability of the SDCD technique.* The change in the balance of the two concordant regions is considerably slower. Figure A3 shows the balances in areas of seen and not-seen as T.d varies: the shift for crossover data is much faster than for concordant data.

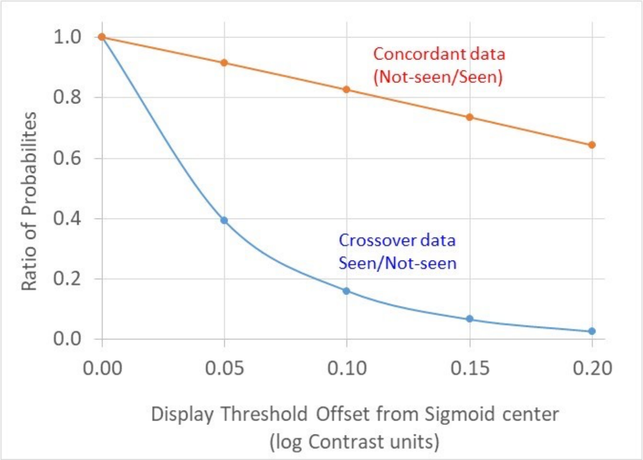

Figure A3

In contrast, the balance of concordant seen and not-seen data changes more slowly as T.d varies, as shown in Figure A3. As T.d shifts by 0.1 log units (Figure A2, top right), the concordant pattern of probability is modified, but in a way unlikely to be easily distinguished visually (Figure A2, bottom right vs. bottom left). An example of crossover vs. concordant data is shown in Figure A4, which shows one slice from Subject 2004: the left plot shows crossover data; the right plot shows concordant data with the same T.d (-0.40 log contrast). In the plot of the concordant data (Figure A4, right) it is difficult to discern reduced sensitivity over the blood vessel, whereas the crossover data show a distinct sensitivity dip, evidenced by a line of not-seen trials.

Adjacent Patches of Visual Field

The above discussion is focused on the statistical properties of data from a single small patch of retina (visual field); however, the same logic applies if we consider adjacent patches of visual field. If T.d is, say, zero everywhere, but the sigmoid for an adjacent patch is shifted left by 0.1 log contrast units, essentially the same change of the balance of areas of the four probability zones will occur as in the top-right diagram in Figure A2. Thus, the rapid shift of balance in the crossover zones will lead to a sharp spatial distinction between adjacent regions of differing sensitivities.

It might seem as if data that are concordant for a particular value of T.d are entirely discarded by the slice technique; however, that is not the case: as the display threshold sweeps through the range of contrast values, the datapoint corresponding to any given trial will show up when that trial result becomes crossover.

When the value of T.d is near the center of the sigmoid for a number of small retinal areas, crossover data for those areas will typically show a mixture of c*rossover seen* in some and c*rossover not-seen* in others. However, as T.d moves, say, up to the highest contrast used, nearly all seen data will become *crossover seen* (and nearly all not-seen data will become *concordant not-seen* and therefore will not be displayed). At the other end of the range of T.d, when it is at the bottom end of the contrast range, nearly all not-seen data will become crossover not-seen data and will be displayed.

The beginning of the first transition can be seen in the top diagrams of Figure A2. Thus, every trial makes an appearance as T.d sweeps through the contrast range. (What seems like a hole in the concordant data at the blind spot, when T.d is at high contrast, reflects the fact that there are never many seen trials in the blind spot.) In summary, the use of crossover data achieves a substantial emphasis of that part of the data that is most informative about the trials with contrast values close to the center of the sigmoid.

It might seem as though there are too few concordant data in Figure A4, given the relative size of the probability areas in Figure A2. (Figure A4 shows every trial from the session in one or the other graph.) The use of algorithms, during an experiment, to determine the next test stimulus at each (nominal) location means that many fewer stimuli are used in the "further reaches" of stimulus contrast (further from the sigmoid centers). That in turn means that testing algorithms substantially reduce the number of concordant trials compared to crossover trials. That is a large experimental (or clinical) gain: using a constant-stimulus approach in the present experiments would require an enormous number of trials—roughly 2,500 in a session that used only 10 contrast levels.

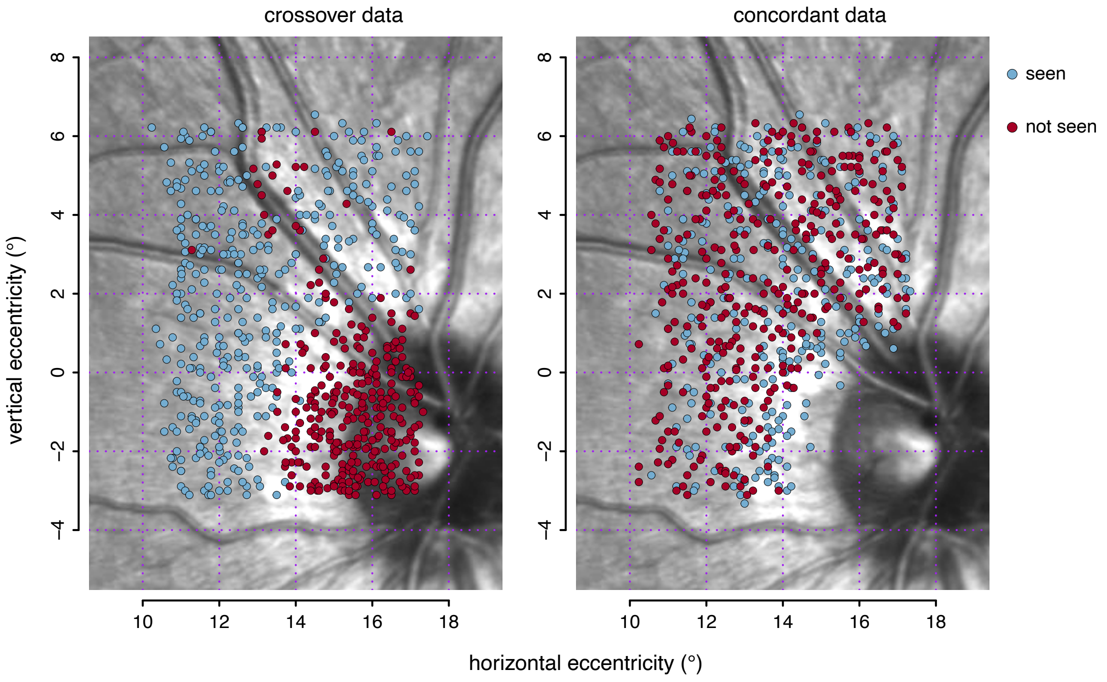

Figure A4. Subject 3, display threshold = -0.50 log Contrast units
